# sxtA4+ and sxtA4- Genotypes Occur Together within Natural *Pyrodinium bahamense* Sub-Populations from the Western Atlantic

**DOI:** 10.3390/microorganisms9061128

**Published:** 2021-05-23

**Authors:** Kathleen Cusick, Gabriel Duran

**Affiliations:** Department of Biological Sciences, University of Maryland Baltimore County, Baltimore, MD 21250, USA; gaduran1@umbc.edu

**Keywords:** bioluminescence, dinoflagellate, *Pyrodinium bahamense*, harmful algal blooms, saxitoxin, *sxtA4*

## Abstract

Saxitoxin (STX) is a secondary metabolite and potent neurotoxin produced by several genera of harmful algal bloom (HAB) marine dinoflagellates. The basis for variability in STX production within natural bloom populations is undefined as both toxic and non-toxic strains (of the same species) have been isolated from the same geographic locations. *Pyrodinium bahamense* is a STX-producing bioluminescent dinoflagellate that blooms along the east coast of Florida as well as the bioluminescent bays in Puerto Rico (PR), though no toxicity reports exist for PR populations. The core genes in the dinoflagellate STX biosynthetic pathway have been identified, and the *sxtA4* gene is essential for toxin production. Using *sxtA4* as a molecular proxy for the genetic capacity of STX production, we examined *sxtA4*+ and *sxtA4*- genotype frequency at the single cell level in *P. bahamense* populations from different locations in the Indian River Lagoon (IRL), FL, and Mosquito Bay (MB), a bioluminescent bay in PR. Multiplex PCR was performed on individual cells with *Pyrodinium*-specific primers targeting the 18S rRNA gene and *sxtA4*. The results reveal that within discrete natural populations of *P. bahamense*, both *sxtA4*+ and *sxtA4*- genotypes occur, and the *sxtA4*+ genotype dominates. In the IRL, the frequency of the *sxtA4*+ genotype ranged from ca. 80–100%. In MB, *sxtA4*+ genotype frequency ranged from ca 40–66%. To assess the extent of *sxtA4* variation within individual cells, *sxtA4* amplicons from single cells representative of the different sampling sites were cloned and sequenced. Overall, two variants were consistently obtained, one of which is likely a pseudogene based on alignment with cDNA sequences. These are the first data demonstrating the existence of both genotypes in natural *P. bahamense* sub-populations, as well as *sxtA4* presence in *P. bahamense* from PR. These results provide insights on underlying genetic factors influencing the potential for toxin variability among natural sub-populations of HAB species and highlight the need to study the genetic diversity within HAB sub-populations at a fine level in order to identify the molecular mechanisms driving HAB evolution.

## 1. Introduction

Saxitoxin is a secondary metabolite produced by several genera of dinoflagellates and cyanobacteria that is better known as a potent neurotoxin due to its detrimental effects on human health. It is the parent molecule in a class of compounds collectively referred to as paralytic shellfish toxins (PSTs), which target voltage-gated ion channels (sodium, potassium, calcium) in humans and can lead to death via respiratory paralysis (as reviewed in [1]). It causes the human illnesses paralytic shellfish poisoning (PSP) and saxitoxin pufferfish poisoning (SPFP) [2], in which shellfish and pufferfish, respectively, ingest the toxic cells and bioaccumulate the toxins within their organs and tissues. STX is produced by microbes from two kingdoms of life inhabiting different aquatic habitats: dinoflagellates (Eukaryota) in marine systems [3,4,5,6], and cyanobacteria (Bacteria) in freshwater systems [7,8], though the biosynthetic pathway is similar between the two groups [9,10].

The gene cluster for STX biosynthesis was first identified in the toxic cyanobacterium *Cylindrospermopsis raciborskii* T3 [11] followed by identification of homologous gene clusters in other cyanobacteria [12,13]. The core genes in STX biosynthesis have more recently been identified in the three marine dinoflagellate genera capable of STX production-multiple *Alexandrium* spp., *Pyrodinium bahamense*, and *Gymnodinium catenatum*–albeit with varying degrees of coverage [14,15]. The first gene in the pathway, *sxtA*, [14], codes for a novel polyketide synthase comprised of four catalytic domains (SxtA1, SxtA2, SxtA3, and SxtA4) [16]. Two different forms of SxtA occur in dinoflagellates: a long transcript yielding all four domains (coded for by the genes *sxtA1*, *2*, *3*, and *4*), and a short transcript lacking *sxtA4* [14]. The gene located at the N-terminal portion of *sxtA* (*sxtA1*), coding for the acyltransferase and phosphopantetheinyl-attachment site domain, is widespread, occurring in both toxic and non-toxic strains of toxic species as well as non-toxic species from other genera [15,17,18]. *SxtA4*, which codes for an amidotransferase [11] appears to be relatively specific for toxin synthesis, as demonstrated by its presence and sequence conservation among numerous toxic strains encompassing all three STX-producing genera and its absence in non-STX-producing species [14,19,20,21].

Multiple examples exist as to both the phenotypic and genetic diversity occurring within monospecific HAB populations and/or blooms. Broad intra-specific variability in multiple traits including toxin production, growth rate, and motility, have been documented among strains (of the same species) isolated from the same water sample or geographic area for various HAB species [22,23,24,25]. In general, a dinoflagellate strain is obtained by establishing a clonal culture, which is derived from the isolation of a single cell from an environmental water sample [24]. Clonal diversity has been shown to be linked to differences in genome size [26], morphology [27], and growth rate [28]. An increase in the use of molecular tools in dinoflagellate population ecology studies have revealed that dinoflagellates are not monoclonal, even in a monospecific bloom [24,29]. High levels of genetic diversity are well-documented among toxic *Alexandrium* dinoflagellate species from the same geographic area, primarily through assessment with microsatellite markers [25,29].

Numerous studies have documented toxin variability in strains isolated from the same geographic area for all three STX-producing genera [22,30,31]. For example, analysis of multiple *A. minutum* strains from the coast of Ireland found strains from southern areas to be toxic but not those from the west coast [22]. Natural populations of *A. tamarense* have been shown to be comprised of a mix of strains, differing in both toxin content and profiles [23]. Studies with a large range of *Gymnodinium* strains demonstrated a high level of intra-population and regional variation in both the presence and amounts of STX congeners and profiles [30]. Additionally, while *P. bahamense* toxic outbreaks are common in Mexico, screening numerous strains from the same area found only one isolate to be a confirmed toxin-producer [31].

The collective findings with microsatellite markers that monospecific dinoflagellate blooms are not monoclonal events coupled with the isolation of both toxic and non-toxic strains of the same species from similar geographic regions underscores the need to examine the genetic capacity for toxin production as a contributor to toxin variability within bloom sub-populations. In the case of the *A. minutum* strains from the south (toxic) and west (non-toxic) coasts of Ireland, the strain differentiation–and by extension toxin potential—was not possible based on common phylogenetic markers, with toxin confirmation achieved only via HPLC analysis [22]. Population genetic studies with *Alexandrium* showed that genetically similar populations (defined via microsatellite markers) differ in toxin production both within and across populations, with natural populations comprised of a mix of strains that differ in both toxin content and profiles [23,25]. Variation in the *sxtA4* genotype in natural populations of dinoflagellate species capable of STX production remains unknown.

*P. bahamense* is a bioluminescent dinoflagellate found in both the Atlantic-Caribbean and the Indo-Pacific [32,33]; toxic outbreaks are well-documented from *P. bahamense* in the Indo-Pacific [5,34,35,36,37,38]. In contrast, *P. bahamense* bloomed in the Indian River Lagoon (IRL), along the east coast of Florida, for years with no known record of STX production. Blooms of *P. bahamense* are the source of the bioluminescence found along both the Florida coast and in the bioluminescent bays in Puerto Rico. However, in the mid-2000s, contaminated seafood outbreaks from multiple states in the US were traced back to Florida, and *P. bahamense* was identified as the source [2,39], marking the first occurrence of toxin production in the Western Atlantic. No genetic data were collected for these sub-populations, and so the molecular mechanisms (*sxt* gene presence and/or regulation) underlying this toxin production remain unknown.

A large gap in our understanding is the factors influencing toxin variability among natural dinoflagellate bloom populations, as both toxic and non-toxic strains of the same species have been isolated from the same geographic locations [31,40]. *SxtA4* is essential for STX biosynthesis [11,14,17,41,42]. Other dinoflagellate genes have been shown to undergo independent gene duplication and shuffling processes [43,44], and in recent years horizontal gene transfer has been demonstrated to be a significant driver in dinoflagellate genome evolution [45,46]. Analysis based primarily on *Alexandrium* isolates indicates that *sxtA4* is lost rather than gained, and that widespread horizontal gene transfer does not occur for this gene [17].

While the core genes in the dinoflagellate STX biosynthetic pathway are known [14,15], how this relates to variability in STX production within natural populations from the same geographic area remains unknown. Recent studies found *sxtA4* gene presence but not transcription in several non-toxic strains of toxic *Alexandrium* spp. [14,17,19,42]. Thus, while “non-toxic” strains may or may not possess the *sxtA4* gene, lack of *sxtA4* is a strong indication of lack of toxicity. In this study, we used *sxtA4* presence within the genome of individual cells as a molecular proxy as to the genetic potential for toxin production among *P. bahamense* cells in a natural population. We use the term “sxtA4+ genotype” to indicate cells in which the sxtA4 gene was detected in the genome; conversely, “sxtA4- genotype” indicates sxtA4 was not found. We examined *sxtA4+* genotype frequency (the proportion of the *sxtA4+* genotype within a population) and *sxtA4* gene variation among single cells within natural *P. bahamense* sub-populations from multiple locations in Florida and Puerto Rico. Our data reveal that both sxtA4+ and sxtA4- genotypes occur within the same sub-population and that frequency of the *sxtA4+* genotype dominates in Florida populations.

## 2. Materials and Methods

### 2.1. sxtA4 Genomic Characterization from P. bahamense Lab Isolate

Genomic DNA and total RNA were extracted from 15 mL (1.5 × 10^5^ cells) of a toxin-producing *P. bahamense* isolate from the IRL using the TRI Reagent kit (Invitrogen, Carlsbad, CA, USA) following manufacturer’s protocol. DNA was diluted 1:10 for subsequent PCRs. RNA was reverse transcribed using the High Capacity RNA-to-cDNA kit (ABI, Waltham, MA, USA), using 1 ug of total RNA. Reactions were performed in a Veriti thermocycler (ThermoFisher, Waltham, MA, USA) under the following conditions: 37 °C for 60 min, followed by 95 °C for 5 min. CDNA was then diluted 1:10 for subsequent PCRs. *SxtA4* DNA was initially amplified using the primers sxt007 and sxt008 (Table 1) with the conditions described by Stuken [14], which yielded a 750 bp product. Additionally, primers were designed based on all publicly-available *P. bahamense sxtA4* sequences in GenBank as of 13 August 2019 to target as nearly a full-length *Pyrodinium*-specific *sxtA4* as possible. This yielded the primer pair sxtA4F1/sxtA4680R (Table 1), which amplified an ca. 680 bp region. PCRs were performed with the ThermoFisher Taq polymerase PCR kit (Waltham, MA, USA) as follows (as final concentrations): 1× KCl (-Mg) buffer, 1.5 mM MgCl_2_, 200 nM each dNTP, 400 nM each forward and reverse primer, 0.625 U Taq DNA polymerase, 2 µL DNA diluted 1:10, and brought to a final volume of 25 µL with nuclease-free water. A touchdown PCR was used that consisted of an initial denaturation at 95 °C for 2 min followed by 2 cycles of 95 °C, 30 s, 58 °C, 15 s, 72 °C, 45 s, and subsequent two cycles with annealing temperatures of 54 °C, 52 °C, 50 °C, 50 °C, 46 °C, and 35 cycles at 44 °C, with a final extension at 72 °C for 7 min. The primer pair sxt007/sxtA4680R was then used to amplify an 815 bp region from both genomic DNA and cDNA. PCRs and thermocycling conditions were as described for the sxtA4F1/680R PCRs. For all PCRs, the resulting amplicons were cloned into the pCR4 vector using the TOPO TA cloning kit (Invitrogen, Carlsbad, CA, USA) and transformed into TOP10 chemically competent Escherichia coli cells following the manufacturer’s instructions. Transformed cells were plated onto Miller’s Luria Broth (LB) plates containing 50 mg mL^−1^ kanamycin (LB_kan50_) and incubated overnight at 37 °C. The resulting transformants were screened via colony PCR using the M13 forward (F) and M13 reverse (R) primers followed by gel electrophoresis to identify those with inserts of the expected size. Colony PCRs were conducted with the ThermoFisher Taq polymerase PCR kit as follows (final concentrations): 1× KCl (-Mg) buffer, 1.5 mM MgCl_2_, 200 nM each dNTP, 200 nM each M13F and M13R primers, 1 U Taq DNA polymerase, and brought to a final volume of 20 uL with nuclease-free water. Thermocycling conditions consisted of an initial denaturation/cell lysis of 95 °C, 3 min, followed by 35 cycles of 95 °C, 15 s, 50 °C, 15 s, 72 °C, 1 min, and a final extension of 72 °C for 7 min. Colonies producing a band of the expected size were inoculated into 2 mL LB_kan50_ broth and grown overnight at 37 °C. The plasmids were isolated using the QIAprep Spin Miniprep kit (Qiagen, Hilden, Germany) and sequenced in both directions using the M13 F and R primers by GeneWiz, Inc. (Germantown, MD, USA). Sequence data were trimmed of primer and vector sequences and evaluated using the BLAST program [47] with comparison against published sequences in GenBank. Sequences from multiple clones were aligned in MEGA7 using ClustalW and manually adjusted.

The 815 bp *stxA4* DNA sequence was aligned with existing *sxtA4* sequences for *Alexandrium* and *Gymnodinium* and used to design additional forward and reverse primers within *sxtA4*. The primer pair sxtA4F1/680R yielded the longest *Pyrodinium*-specific *sxtA4* sequence and so was then optimized for use in a single-cell multiplex PCR assay with *P. bahamense*-specific 18S rRNA gene primers. The sxtA4166F primer targeted a conserved region within *sxtA4* and so was used in subsequent nested PCRs and sequencing reactions (described below).

### 2.2. sxtA4 Genotype Analysis from Single Cells

#### 2.2.1. Sample Collection

**Figure 1:**
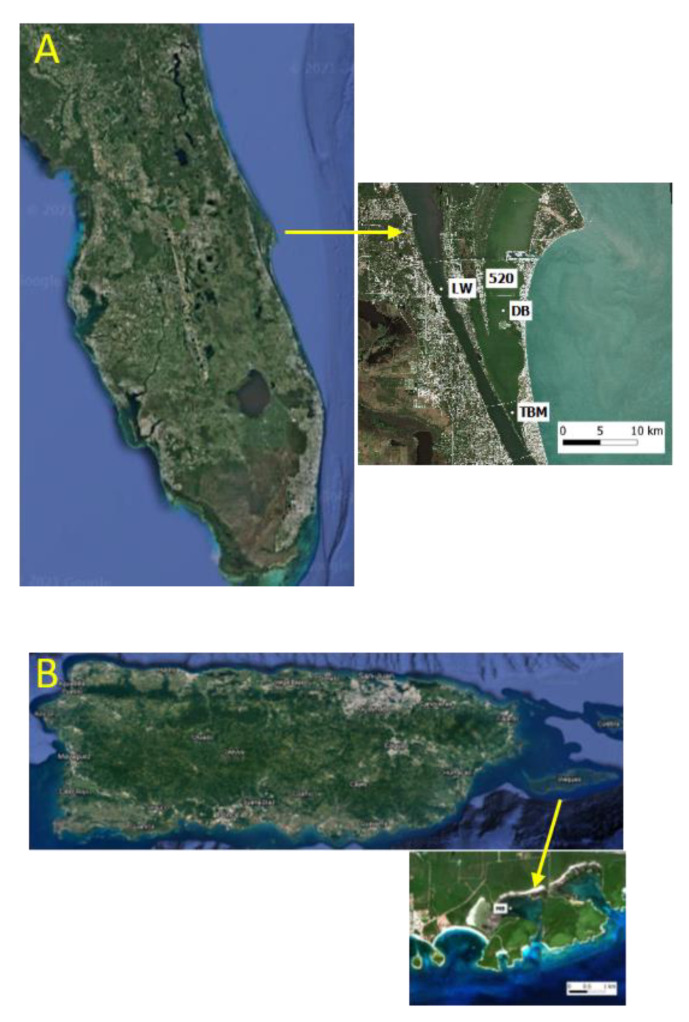
Map depicting sampling sites in (**A**) Indian River Lagoon, FL and (**B**) Mosquito Bay in Vieques, Puerto Rico. Florida sampling sites: TBM = Telemar Bay Marina, DB = Diamond Bay, LW = Lee Wenner Park, 520 = 520 Bridge. MB (Vieques map) = Mosquito Bay.

Whole water samples were collected from multiple locations in the IRL and Mosquito Bay, PR, from July–September 2019 and 2020 from the surface to a depth of 0.5 m (Figure 1). Samples from the IRL were collected onto 30-micron mesh and the biomass collected on the mesh rinsed into a sterile 1 L bottle using filtered water. In Mosquito Bay, whole-water grab samples were collected without size fractionation. Samples were transported at ambient temperature back to the laboratory for processing.

#### 2.2.2. Cell Isolation and Lysis

Single cells were isolated for lysis and subsequent multiplex PCR using a slight modification of a protocol described previously [48]. Briefly, an aliquot (ca. 1–3 mL) of the environmental sample water was placed in a petri dish and viewed with an inverted light microscope. *P. bahamense* cells were identified under 400× magnification based on morphology [49]. At all sampling sites, *P. bahamense* was the dominant dinoflagellate species. Individual cells were isolated with a sterile glass micropipette under 100× magnification, washed twice in sterile HPLC water, and placed in sterile 200 μL PCR tubes. Samples were stored at −20 °C until DNA extraction. Prior to DNA extraction, the final volume of water in each tube was brought to 20 uL with nuclease-free water. DNA extraction consisted of five consecutive freeze-thaw cycles alternating between baths of a dry ice/ethanol slurry and heating to 100 °C. Tubes were centrifuged briefly prior to PCR.

#### 2.2.3. Multiplex PCR

Multiplex PCR was performed on single cells using *Pyrodinium*-specific primers targeting the 18S rRNA gene developed previously [48] and the sxtA4F1/stxA4680R primers. The single-cell multiplex PCR was first optimized on single cells from lab cultures of the toxin-producing *P. bahamense* isolate from the IRL using the same methods for cell isolation and washing, lysis, and PCR as for environmental samples. While all reagents of the PCR were optimized, those which significantly improved the reaction included an increase in final magnesium (2.5 mM) and dNTP (0.3 mM) concentrations as well as a decreased concentration of the 18S rRNA gene primers (200 nM final concentration) in comparison to the sxtA4 F1/680R primers (400 nM final concentration). All single cells from the toxic lab isolate that yielded an 18s rRNA gene amplicon also yielded the 680 bp *sxtA4* amplicon (data not shown), indicating the ability of both primer sets to bind to and amplify their respective targets when used in the same PCR. Following the optimization, PCRs were performed on single cells from environmental samples. Single cell multiplex PCRs utilized the GoTaq PCR Core System I kit (Promega, Madison, WI, USA) and consisted of the following (as final concentrations): 1× Colorless GoTaq Flexi buffer, 2.5 mM MgCl_2_, 0.3 mM dNTP mix, 200 nM each Pcomp370F and Pcomp1530R, 400 nM each sxtA4F1 and sxtA4680R, 3.7 U GoTaq DNA polymerase, and brought to a final volume of 50 µL with 30 µL nuclease-free water. Thermocycling conditions consisted of an initial denaturation at 95 °C for 3 min followed by 45 cycles of 95 °C for 30 s; 57 °C for 15 s; 72 °C for 1 min; and a final extension at 72 °C for 7 min. Multiplex PCRs were also performed on a subset of samples (ca. 40 single cells) with the *sxtA4* primer pair sxt007/sxt008, as this primer pair has been used in all *sxtA4* analyses to date for *Alexandrium*, *Gymnodinium*, and *Pyrodinium*, and thus appears to be conserved across species. Products were visualized on 1% E-Gel EX agarose gels with the E-Gel Power Snap Electrophoresis System (Invitrogen). The 18S rRNA gene served as a positive control to confirm the presence of the cell in the tube and that reactions were not inhibited by potential contaminants. Only samples yielding the 18S rRNA gene amplicon were included in *sxtA4* genotype frequency analysis.

Samples yielding products indicative of both the 18S rRNA gene (1200 bp) and *sxtA4* (680 bp) were further screened by one (or more) additional means to confirm *sxtA4* amplicon specificity. These included: (1) the multiplex PCRs were PCR purified using the Wizard S/V Gel and PCR Clean-Up System (Promega, Madison, WI) and sequenced (as two separate samples) with the internal primer sxtA4166F and the Pcomp350F primer. The sequencing protocol was modified so that the amount required for each product was doubled; (2) bands corresponding to the 18S rRNA gene and *sxtA4* amplicons were gel-purified using the Wizard S/V Gel and PCR Clean-Up System and sequenced with the Pcomp350 F and sxtA4166F primers, respectively; (3) a nested PCR was performed in which the original multiplex PCR product was diluted 1:5 and 2 uL used as the template for a PCR using the sxtA4166F internal primer and 680R. These PCRs utilized the GoTaq PCR Core System I and consisted of the following (as final concentrations): 1× Colorless GoTaq Flexi buffer, 1.5 mM MgCl_2_, 0.1 mM dNTP mix, 400 nM each sxtA166F and sxtA4680R, 0.625 U GoTaq DNA polymerase, and brought to a final volume of 25 µL with nuclease-free water. Thermocycling conditions consisted of 95 °C for 3 min, followed by 35 cycles of 95 °C, 30 s, 57 °C, 15 sec, 72 °C, 40 s, and a final extension at 72 °C for 7 min. The resulting product was visualized with gel electrophoresis; with this primer set, an amplicon of ca. 500 bp was indicative of *sxtA4*. These samples were then PCR purified and sequenced with 166F to confirm *sxtA4* specificity.

Samples in which only a band indicative of the 18S rRNA gene was amplified were PCR purified and sequenced with Pcomp350F to confirm *P. bahamense* specificity. For this, 20 uL of the initial multiplex was cleaned using the Wizard PCR Clean-Up kit. Subsequent PCRs were then performed on the initial multiplex with the primer sets 166F/680R and F1/680R to explore whether variants were missed using the *Pyrodinium*-specific sxtA4F1 (see details in Supplementary Materials). As the initial multiplex PCR was performed in the same tube that contained the single cell, the genomic DNA, and thus template, was still present in the tube for these PCRs.

To confirm their identity, the obtained partial sequences of *sxtA4* and the 18S rRNA gene were queried against the GenBank nr database using standard BLASTN 2.2.26+ [50].

To examine the extent of *sxtA4* gene variation within individual cells from different populations, the *sxtA4* amplicon (yielded with the 166F/680R primer pair for an amplicon length of ca. 500 bp) from a small subset of individual cells (one representative of each site in the IRL plus MB) was cloned, and a minimum of 10 clones from each sequenced. Amplicons were cloned into the pCR-4 vector using the TOPO TA cloning kit (Invitrogen), transformed into chemically competent TOP10 *E. coli* cells following the manufacturer’s instructions for kanamycin selection, and sequenced using the M13 forward and reverse primers by GeneWiz (Germantown, MD, USA).

Sequence data were trimmed of primer and vector sequences and initially queried against the GenBank nr database using standard BLASTN 2.2.26+ [50]. All subsequent analysis was performed using MEGA (Molecular Evolutionary Genetics Analysis) v7. The phylogenetic relationship among sequences in relation to geographic location (as defined by the four sampling sites) was examined to assess whether sequences were specific for a location. *SxtA4* clone sequences obtained from individual cells representative of the four sampling sites as well as *Alexandrium* spp. *sxtA4* sequences retrieved from GenBank were used in the analysis. The model that best fit the data was the Tamura 3-parameter (T92) and so was used to generate Neighbor Joining and minimal evolution trees. Both trees produced identical results. The sequence data were also compared to existing *Pyrodinium sxtA4* sequences in GenBank (MN431957.1, a 358bp fragment, designated as var. *compressum* from Sepanggar Bay, Malaysia; and a 681 bp sequence, from TSA GBXF01000001.1, also designated as var. *compressum*).

Sequences obtained in this study have been have been deposited into GenBank with the following accession numbers: MZ234664-MZ234695.

## 3. Results and Discussion

### 3.1. SxtA4 Genomic Characterization

**Figure 2:**
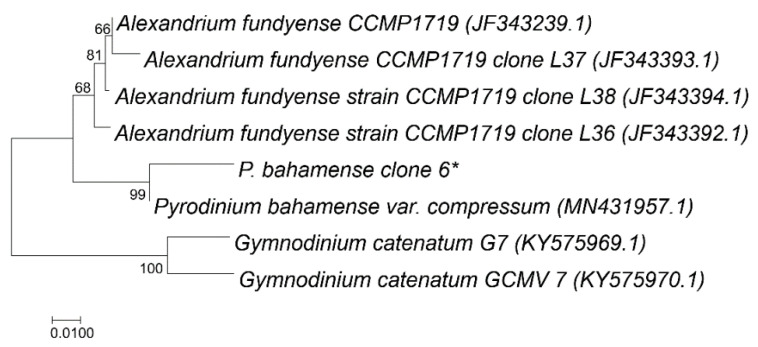
Neighbor Joining tree of *sxtA4* sequences. Representative *Alexandrium*, *Pyrodinium*, and *Gymnodinium sxtA4* sequences were downloaded from GenBank and aligned and trimmed in MEGA 7. Percentages from the bootstrap test (500 replicates) are displayed next to the branches. The rate variation among sites was modeled with a gamma distribution (shape parameter = 0.32). The analysis encompassed 8 nucleotide sequences. All positions containing gaps and missing data were eliminated. There were a total of 575 positions in the final dataset. Evolutionary analyses were conducted in MEGA7. * indicates *P. bahamense sxtA4* sequence from this study.

The ca 815 bp *sxtA4* sequence amplified here is the longest to date for *P. bahamense*. Genomic DNA sequences were highly similar among clones, ranging from 96–100%. This was also the case for the cDNA clones, albeit with even greater similarity (99–100%) and overall, the two types of sequences showed a high degree of similarity (Table 2). Alignment of sequences amplified from genomic DNA demonstrated minimal sequence diversity, with 29 base substitutions. Alignment of sequences derived from cDNA showed even less diversity, with only seven base substitutions. This is in keeping with the trend recorded for *Alexandrium*, in that the overall percent similarity was higher for cDNA copies than gDNA copies, indicating that only certain copies are transcribed [14,19]. Phylogenetic analysis demonstrated *sxtA4* for *P. bahamense* from the IRL clustered with *sxtA4* from a toxic Indo-Pacific strain, and these sequences grouped with *Alexandrium*, with *Gymnodinium* forming a separate cluster (Figure 2).

In comparing the gDNA sequences of the *P. bahamense* lab isolate, two main variants occurred, as defined by consistent base substitutions at 29 sites (i.e., base 182, 4 clones possess “G”, the other 12 clones a “C”; base 234, the same 4 clones possess “T”, the other 12 a “C”). This was in addition to 27 sites in which the base of an individual clone differed from the others. It has been suggested that harboring multiple, slightly different genomic copies of *sxtA4* may be the underlying basis for the diversity in STX congener profiles and toxicity within *Alexandrium*. The *P. bahamense* lab isolate consistently yields a STX profile of two congeners: STX and GTX-5 (Table S1), even when grown under various conditions.

### 3.2. sxtA4+/sxtA4- Genotypes in Natural P. bahamense Sub-Populations

Multiplex PCR screening of individual cells revealed that both *sxtA4+* and *sxtA4-* genotypes exist among natural *P. bahamense* populations, with the *sxtA4+* genotype defined by the amplification of *sxtA4* in individual cells. The data to date show that among all sites, the *sxtA4+* genotype dominates, regardless of sample size (Table 3). However, it is worth noting that sequence data were derived from summer (June–September) samplings. In the IRL, *P. bahamense* blooms during this period. *P. bahamense* is present as both bloom and non-bloom events in the IRL. The northern region has particularly long water residence times, and is prone to intense blooms [51,52,53]. Major blooms of *P. bahamense* in the IRL are defined as > 100 cells/mL; a several-year study on *P. bahamense* distribution in the IRL recorded densities up to 776 cells/mL [54]. However, since that time, greater cell densities, as well as their locations within the northern IRL, have been observed (Cusick pers. observ.) Microscopic observations of water samples collected at the various sites in the IRL showed *P. bahamense* to be the dominant cell type at all. In the bioluminescent bay in Puerto Rico, *P. bahamense* was also the dominant cell type. While not as well-studied as the IRL, a recent one-year study in Mosquito Bay recorded an overall average of ca. 27 cells mL^−1^, with abundance ranging from 0–244 cells mL^−1^ [55]. A primary difference among sites is that *P. bahamense* is present in Mosquito Bay year-round, while it (typically) disappears over the fall and winter months in the IRL.

**Figure 3:**
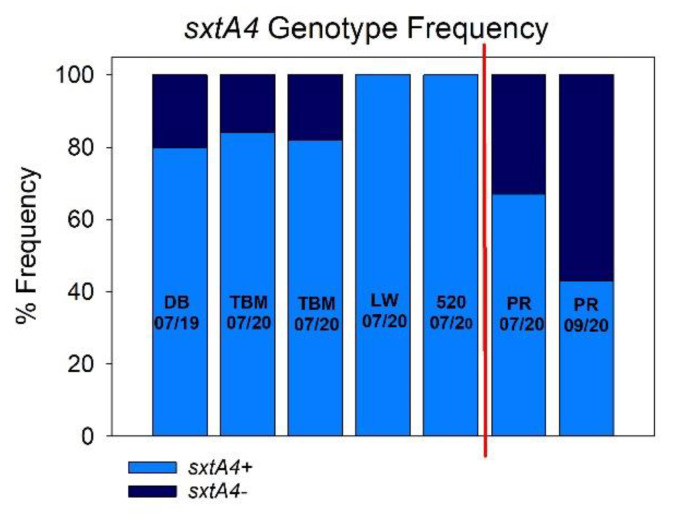
Percent *sxtA4* genotype frequency among sites. Multiplex PCR was performed on individual *P. bahamense* cells collected from multiple sites in the Indian River Lagoon (IRL, Florida) and Mosquito Bay, a bioluminescent bay in Puerto Rico. Cells yielding the *sxtA4* amplicon were defined as “sxtA4+”; cells in which *sxtA4* was not detected were defined as “*sxtA4*-“. Text and numbers in each bar represent sampling site and date: DB = Diamond Bay (IRL); TBM = Telemar Bay Marina (IRL); LW = Lee Wenner Park (IRL); 520 = 520 Bridge (IRL). Vertical red line in graph separates Florida and Puerto Rico (PR) sampling sites.

In the IRL, the frequency of the *sxtA4+* genotype ranged from ca. 80–100% (Table 3, Figure 3). Sampling at Diamond Bay (July 2019) showed the *sxtA4+* genotype to occur at 80% frequency. Comparable numbers were obtained for samplings at Telemar Bay Marina in 2020 in both June and July, with the *sxtA4+* genotype occurring at frequencies of 81% and 84%, respectively. The *sxtA4+* genotype occurred at a frequency of 100% in populations from sites LW and 520. The *sxtA4+* genotype occurred at a much lower frequency in Mosquito Bay (Puerto Rico) populations, at ca 67% in July and decreasing to ca 40% in Sept (Figure 3). While these data must be interpreted with caution due to the low sample size, these are the first data demonstrating the presence of *sxtA4* in *P. bahamense* populations in bioluminescent bays in Puerto Rico.

The correlation between *sxtA4* genomic presence and active toxin production within both Florida and Puerto Rico sub-populations is not known. *P. bahamense* is part of a routine state HAB monitoring program (Florida Fish and Wildlife Conservation Commission) in Florida. Following the initial determination (2002–2004) of *P. bahamense* as the source of saxitoxin in pufferfish and shellfish harvested from the IRL, state agencies established the PSP Biotoxin Contingency Program to monitor saxitoxin in shellfish. Since that time, 39 closures of shellfish harvesting areas have occurred based on STX levels exceeding the international standard action level. However, natural *P. bahamense* populations are not routinely measured for STX production. *P. bahamense* populations in Puerto Rico have not been measured for toxicity and are presumed to be non-toxic (pers. comm., VCHT staff). Overall, this study consisted of a small sample size, and more widespread sampling would be needed to draw conclusions about the relative proportions of *sxtA4* genotypes in the areas sampled and the relationships of these results to the STX levels in the regions.

### 3.3. Gene Variants

Direct sequencing of *sxtA4* amplicons from single cells indicated multiple variants, evidenced by base substitutions that consistently occurred in an ca. 200 bp region (data not shown). Additionally, two distinct variants were consistently obtained from most cells, one of which displayed a 15 bp deletion.

Further testament as to *sxtA4* diversity within *P. bahamense* was evidenced by samples in which *sxtA4* was not initially detected. Single cells which yielded only an 18S rRNA gene amplicon in the initial multiplex were then screened with two additional PCRs: (1) the nested PCR using the 166F/680R primer set that targeted a conserved region of the *sxtA4* gene and (2) a PCR with only the sxtA4 F1/680R primers. A small subset of these samples yielded an amplicon indicative of *sxtA4* with the 166F/680R primers. Sequencing of these 500 bp amplicons confirmed *sxtA4*. Amplification with the conserved primer (166F) but not the *Pyrodinium*-specific (F1) suggests sequence variation in this region (F1 is located 165 bp upstream of 166F) of *P. bahamense*. In comparing *sxtA4* sequences available in GenBank that encompassed this region (all *Alexandrium* spp. and a single *Pyrodinium*) and gDNA and cDNA sequences of the toxic lab isolate, the F1 primer displayed sequence variation in *Alexandrium*, while the 166F primer was conserved across *Pyrodinium* and *Alexandrium* spp (see sequence alignments in Supplementary Materials).

**Figure 4:**
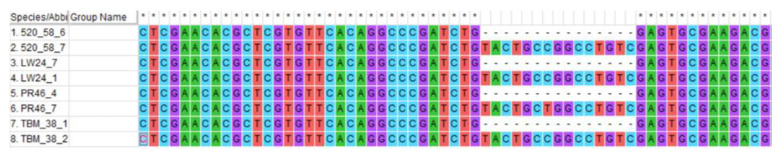
Two distinct variants were recovered from individual cells at all sampling sites, defined by a 15 bp deletion in one of the variants.

To assess the extent of *sxtA4* variation within individual cells, the *sxtA4* amplicons from a small subset of single cells representative of the different sampling sites were cloned and sequenced. As with the direct sequencing of *sxtA4* PCR amplicons, two variants were consistently obtained from clones of all samples, with one of the variants displaying the 15 bp deletion (Figure 4). The *sxtA4* DNA sequences amplified and cloned from single cells were aligned with the cDNA sequences from the lab isolate. The 15 bp region was present in the cDNA copies of the lab isolate, indicating that this variant is likely a pseudogene. This was found to be the case with *A. fundyense* strains, in which a 63 bp deletion was found in some gDNA sequences but not the corresponding cDNA sequences [17].

*SxtA4* clone sequences from Puerto Rico showed two general variants, defined by the presence or absence of the 15 bp stretch. Overall, the percent identity among sequences ranged from 96–100%; minimal (2–5) base substitutions occurred among clones from the same general variant. *SxtA4* clones from the TBM site also showed the two variants. A greater diversity of sequences were obtained, with the percent identity spanning 91–98%. Both sequence variants were also obtained from site LW, and percent identity ranged from 95–99%. The two sxtA4 variants were also recovered from clones from 520, with percent identity ranging from 95–99%.

**Figure 5:**
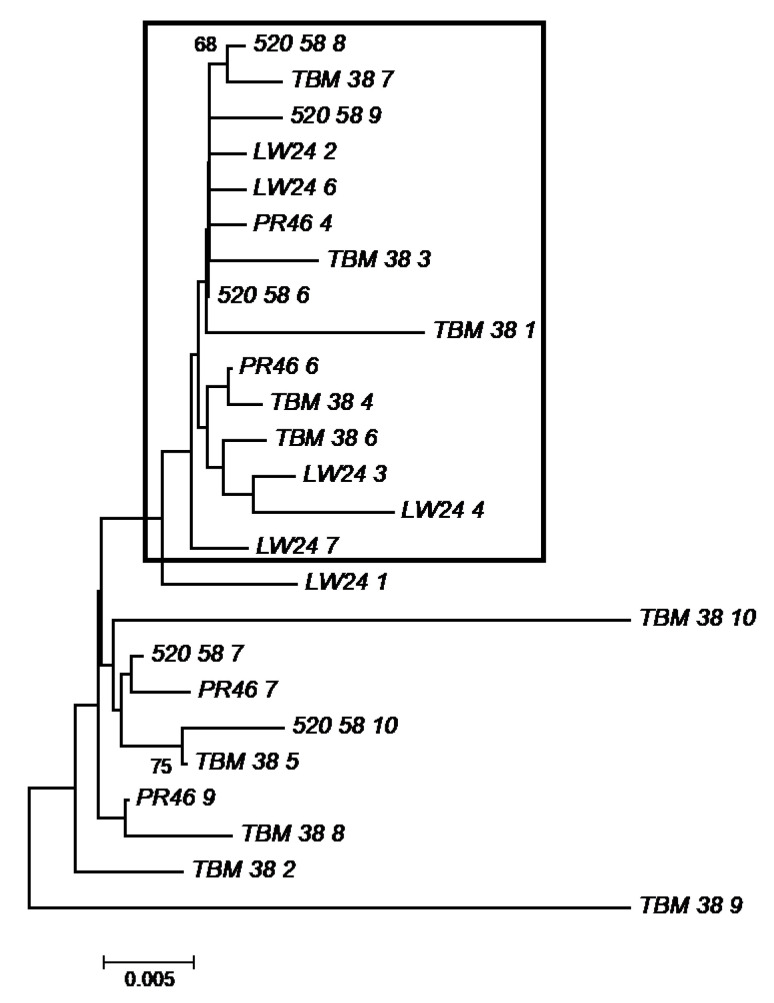
Neighbor Joining tree of *sxtA4* sequences obtained from clones of single cells of *P. bahamense* from the representative sampling sites. Percentages from the bootstrap test (500 replicates) are shown next to the branches. The Tamura 3-parameter method best fit the data and so was used to compute the evolutionary distances. Twenty-five nucleotide sequences were included in the analysis. All ambiguous positions were removed for each sequence pair. There were a total of 517 positions in the final dataset. Evolutionary analyses were conducted in MEGA7. Nomenclature is as follows: Sampling site_cell_clone number. Note that for each site, the “cell” number is the same, as all sequences from that site were derived from the same cell. DB = Diamond Bay (IRL); TBM = Telemar Bay Marina (IRL); LW = Lee Wenner Park (IRL); 520 = 520 Bridge (IRL); PR = Mosquito Bay in Puerto Rico. Sequences with the 15 bp deletion are boxed.

A neighbor-joining tree constructed with the cloned *sxtA4* amplicons from individual cells showed sequences formed two clusters; however, these clusters were not defined by sampling site/population but rather by the presence/absence of the 15 bp region (Figure 5). Comparison of the two representative sequences from the single cell clones with existing *Pyrodinium sxtA4* sequences showed the sequence variant not lacking the 15 bp segment was 99% identical to existing *P. bahamense sxtA4* sequences, and the variant with the 15 bp deletion was 95–96% identical (Table S2). This indicates the gene is conserved across populations, though this must be interpreted with caution due to the limited number of sequences and the length of the sequences.

## 4. Conclusions

*P. bahamense* was previously classified as a single species with two varieties based on biochemical and morphological differences [56]. The Indo-Pacific variety was designated ‘compressum’, and the Atlantic-Caribbean variety ‘bahamense’. However, both “varieties” have since been found in the same geographic locations, and one of the key distinguishing differences, the lack of toxin production by var. *bahamense*, has since been disproven. The varietal designation has been removed based on a recent reinvestigation of morphological attributes, and the suggestion that the presence of both varieties within the same plankton sample is likely the occurrence of different life stages [57]. Large subunit rRNA gene sequence analysis indicated the existence of Indo-Pacific and Atlantic-Caribbean ribotypes, leading to the suggestions that *P. bahamense* is a species complex. A previous study showed 18S rRNA gene sequences of *P. bahamense* from multiple locations in the IRL, Puerto Rico, and the Indo- Pacific to be nearly identical and forming a single cluster [48]. Comparison of 18S rRNA gene sequences from *sxtA4+* and *sxtA4-* genotype cells in this study were compared to existing 18S rRNA gene sequences from the Indo-Pacific and Atlantic-Caribbean. Not surprising, all sequences were 99.6–100% identical (SI).

A previous study by our group showed, at the single cell level, that two different luciferase (*lcf*, responsible for bioluminescence) variants were consistently recovered from *P. bahamense* populations in both Florida and Puerto Rico [48]. *Lcf* codes for a single polypeptide comprised of three homologous domains (“D1”, “D2”, “D3”) [58,59]. The individual domains among species (i.e., D1 *A. tamarense*, D1 *A. affine*) are more similar than among the three different domains of the same species. The sequences amplified from individual cells from Florida and Puerto Rico formed two distinct clusters defined by a set of non-synonymous substitutions. Only one of the two types of *lcf* sequence, but not both, was recovered from each individual cell. Both types of *lcf* sequences were obtained from all sampling sites. Primers selectively amplified domain 3 (D3), indicating the two *P. bahamense lcf* sequences represented gene variants rather than amplification of the different domains. The two *P. bahamense* sequences are 87% similar. The variation between the two *P. bahamense lcf* sequences is much greater than that of bioluminescent species with known gene variants (i.e., *Pyrocystis lunula* possesses LcfA and LcfB, with 97% amino acid similarity). The significance underlying this variation remains unknown.

The results obtained here demonstrate that within small discrete natural populations (i.e., 3 L of surface water) of *P. bahamense* there exist both *sxtA4+* and *sxtA4-* genotypes. Correlation of *lcf* variant with *sxtA4* genotype may provide insights as to a potential *Pyrodinium* species complex. The next step will be determining how and if *sxtA4+/-* frequencies change over time, and the environmental factors driving these fluctuations. Understanding the factors driving bloom diversity and evolution is essential to understanding bloom dynamics and success over both space and time. This is the first study, to our knowledge, to examine the genetic potential for toxin production within natural dinoflagellate bloom sub-populations, which is critical for understanding bloom ecology and evolution.

## Figures and Tables

**Table 1:** List of primers.

Primer	Sequence	Source
PyrosxtA4F1	AACGACATGAAGCAGCTCGA	this study
PyrosxtA4R680	CTAGATGGGGTACCACATAG	this study
SxtA4166F	CATGGCTGCGGCGTTCTTG	this study
Pcomp370F	AAATTACCCAATCCTGACACT	48
Pcomp1530R	CTGATGACTCAGGCTTACT	48
sxt007	ATGCTCAACATGGGAGTCATCC	14
sxt008	GGGTCCAGTAGATGTTGACGATG	14

**Table 2:** Percent similarity between genomic and cDNA *sxtA4* sequences for *P. bahamense*.

Species	No. cDNA Sequences	% Similarity	No. DNA Sequences	% Similarity
*P. bahamense IRL*	8	99.1–100	14	96.0–100
*A. fundyense ^1^ CCMP1719*	12	96.2–99.4	10	91.7–100
*A. fundyense A8 ^1^*	7	93.5–100	19	77.5–100
*A. fundyense E4 ^1^*	19	97.0–100	40	76.8–100
*A. pacificum ACCC01 ^1^*	4	96.0–100	5	91.0–100

^1^ Alexandrium data [14,17] are provided for comparison.

**Table 3:** *sxtA4*+ genotype frequencies from sites in Florida (IRL) and Puerto Rico. ^1^ indicates number of isolated cells yielding an 18S rRNA gene amplicon. ^2^ Number of isolated cells yielding the 18S rRNA gene amplicon in which *sxtA4* was detected.

Sampling Site	Date	18S ^1^	*sxtA4 ^2^*	*sxtA4+* Frequency
Diamond Bay (IRL)	7/15/2019	10	8	80%
TBM (IRL)	6/15/2020	64	54	84%
TBM (IRL)	7/20/2020	44	36	82%
Lee Wenner (IRL)	7/20/2020	30	30	100%
520 Bridge (IRL)	7/20/2020	37	37	100%
Mosquito Bay (PR)	7/8/2020	9	6	67%
Mosquito Bay (PR)	9/15/2020	7	3	43%

## Data Availability

Sequences described in this study have been submitted to GenBank.

## Supplementary Materials

The following are available online at https://www.mdpi.com/article/10.3390/microorganisms9061128/s1, Detailed methods on additional screenings of *P. bahamense* cells following the initial multiplex PCR; sequence comparisons of *Pyrodinium* and *Alexandrium* illustrating sequence variability in sxtA4F1 and 166F primer binding sites; Table S1 and Methods for Detection of STX congeners in *P. bahamense* toxic lab isolate; Table S2: Percent Identity among *sxtA4 Pyrodinium bahamense* clones; representative 18S rRNA gene sequences used in *P. bahamense* analysis.

## References

[1] Cusick K.D., Sayler G.S. (2013). An overview on the marine neurotoxin saxitoxin: Genetics, molecular targets, methods of detection, and ecological functions. Mar. Drugs.

[2] Landsberg J.H., Hall S., Johannessen J.N., White K.D., Conrad S.M., Abbott J.P., Flewelling L.J., Richardson R.W., Dickey R.W., Jester E.L.E. (2006). Saxitoxin puffer fish poisoning in the United States, with the first report of Pyrodinium bahamense as the putative toxin source. Environ. Health Persp..

[3] Anderson D.M., Kulis D.M., Sullivan J.J., Hall S., Lee C. (1990). Dynamics and physiology of saxitoxin production by the dinoflagellates Alexandrium spp. Mar. Biol..

[4] Hallegraeff G.M., Steffensen D.A., Wetherbee R. (1988). Three estuarine Australian dinoflagellates that can produce paralytic shellfish toxins. J. Plankton Res..

[5] Harada T., Oshima Y., Kamiya H., Yasumoto T. (1982). Confirmation of paralytic shellfish toxins in the dinoflagellate pyrodinium-bahamense var. compressa and bivalves in Palau. Bull. Jpn. Soc. Sci. Fish..

[6] Oshima Y., Hasegawa M., Yasumoto T., Hallegraeff G., Blackburn S. (1987). Dinoflagellate Gymnodinium catenatum as the source of paralytic shellfish toxins in tasmanian shellfish. Toxicon.

[7] Carmichael W.W., Evans W.R., Yin Q.Q., Bell P., Moczydlowski E. (1997). Evidence for paralytic shellfish poisons in the freshwater cyanobacterium Lyngbya wollei (Farlow ex Gomont) comb. Nov. Appl. Environ. Microbiol..

[8] Negri A.P., Jones G.J. (1995). Bioaccumulation of paralytic shellfish poisoning (PSP) toxins from the cyanobacterium Anabaena circinalis by the freshwater mussel Alathyria condola. Toxicon.

[9] Shimizu Y. (1993). Microalgal metabolites. Chem. Rev..

[10] Shimizu Y. (1996). Microalgal metabolites: A new perspective. Annu. Rev. Microbiol..

[11] Kellmann R., Mihali T.K., Jeon Y.J., Pickford R., Pomati F., Neilan B.A. (2008). Biosynthetic intermediate analysis and functional homology reveal a saxitoxin gene cluster in cyanobacteria. Appl. Environ. Microbiol..

[12] Mihali T.K., Kellmann R., Neilan B.A. (2009). Characterisation of the paralytic shellfish toxin biosynthesis gene clusters in Anabaena circinalis AWQC131C and Aphanizomenon sp. NH-5. BMC Biochem..

[13] Mihali T.K., Carmichael W.W., Neilan B.A. (2011). A putative gene cluster from a Lyngbya wollei bloom that encodes paralytic shellfish toxin biosynthesis. PLoS ONE.

[14] Stuken A., Orr R.J.S., Kellmann R., Murray S.A., Neilan B.A., Jakobsen K.S. (2011). Discovery of nuclear-encoded genes for the neurotoxin saxitoxin in dinoflagellates. PLoS ONE.

[15] Hackett J.D., Wisecarver J.H., Brosnahan M.L., Kulis D.M., Anderson D.M., Bhattacharya D., Plumley F.G., Erdner D.L. (2013). Evolution of saxitoxin synthesis in cyanobacteria and dinoflagellates. Mol. Biol. Evol..

[16] Kellmann R., Michali T.K., Neilan B.A. (2008). Identification of a saxitoxin biosynthesis gene with a history of frequent horizontal gene transfers. J. Mol. Evol..

[17] Murray S.A., Diwan R., Orr R.J.S., Kohli G.S., John U. (2015). Gene duplication, loss, and selection in the evolution of saxitoxin biosynthesis in alveolates. Mol. Phylogenetics Evol..

[18] Wang H., Kim H., Ki J.-S. (2020). Transcriptome survey and toxin measurements reveal evolutionary modification and loss of saxitoxin biosynthesis genes in the dinoflagellates Amphidinium carterae and Prorocentrum micans. Ecotoxicol. Environ. Saf..

[19] Murray S.A., Wiese M., Stuken A., Brett S., Kellmann R., Hallegraeff G., Neilan B.A. (2011). SxtA-based quantitative molecular assay to identify saxitoxin-producing harmful algal blooms in marine waters. Appl. Environ. Microbiol..

[20] Orr R.J.S., Stuken A., Murray S.A., Jakobsen K.S. (2013). Evolutionary acquisition and loss of saxitoxin biosynthesis in dinoflagellates: The second "core" gene, sxtG. Appl. Environ. Microbiol..

[21] Murray S.A., Hoppenrath M., Orr R.J.S., Bolch C., John U., Diwan R., Yauwenas R., Harwood T., de Salas M., Neilan B. (2014). Alexandrium diversaporum sp. nov., a new non-saxitoxin producing species: Phylogeny, morphology and sxtA genes. Harmful Algae.

[22] Touzet N., Franco J.M., Raine R. (2007). Characterization of nontoxic and toxin-producing strains of Alexandrium minutum (Dinophyceae) in Irish coastal waters. Appl. Environ. Microbiol..

[23] Alpermann T.J., Tillmann U., Beszteri B., Cembella A.D., John U. (2010). Phenotypic variation and genotypic diversity in a planktonic population of the toxigenic marine dinoflagellate Alexandrium tamarense (Dinophyceae). J. Phycol..

[24] Menden-Deuer S., Montalbano A.L. (2015). Bloom formation potential in the harmful dinoflagellate Akashiwo sanguinea: Clues from movement behaviors and growth characteristics. Harmful Algae.

[25] Anderson D.M., Alpermann T.J., Cembella A.D., Collos Y. (2012). Masseret E, Montresor M. The globally distributed genus Alexandrium: Multifaceted roles in marine ecosystems and impacts on human health. Harmful Algae.

[26] Whittaker K.A., Rignanese D.R., Olson R.J., Rynearson T.A. (2012). Molecular subdivision of the marine diatom Thalassiosira rotulain relation to geographic distribution, genome size, and physiology. BMC Evol. Biol..

[27] Saravanan V., Godhe A. (2010). Genetic heterogeneity and physiological variation among seasonally separated clones of Skeletonema marinoi (Bacillariophyceae) in the Gullmar Fjord, Sweden. Eur. J. Phycol..

[28] Rynearson T.A., Armbrust E.V. (2004). Genetic differentiation among populations of the planktonic marine diatim Ditylum brightwellii (Bacillariophyceae). J. Phycol..

[29] Richlen M.L., Erdner D.L., McCauley L.A.R., Libera K., Anderson D.M. (2012). Extensive genetic diversity and rapid population differentiation during blooms of Alexandrium fundyense (Dinophyceae) in an isolated salt pond on Cape Cod, MA, USA. Ecol. Evol..

[30] Hallegraeff G.M., Blackburn S.I., Doblin M.A., Bolch C.J.S. (2012). Global toxicology, ecophysiology and population relationships of the chainforming PST dinoflagellate Gymnodinium catenatum. Harmful Algae.

[31] Morquecho L. (2019). Pyrodinium bahamense, one the most significant harmful dinoflagellates in Mexico. Front. Mar. Sci..

[32] Phlips E.J., Badylak S., Christman M., Wolny J., Brame J., Garland J., Hall L., Hart J., Landsberg J., Lasi M. (2011). Scales of temporal and spatial variability in the distribution of harmful algae species in the Indian River Lagoon, Florida, USA. Harmful Algae.

[33] Usup G., Ahmada A., Matsuoka K., Lim P.T., Leaw C.P. (2012). Biology, ecology and bloom dynamics of the toxic marine dinoflagellate Pyrodinium bahamense. Harmful Algae.

[34] Montojo U.M., Sakamoto S., Cayme M.F., Gatdula N.C., Furio E.F., Relox J.J.R., Shigeru S., Fukuyo Y., Kodama M. (2006). Remarkable difference in accumulation of paralytic shellfish poisoning toxins among bivalve species exposed to Pyrodinium bahamense var. compressum bloom in Masinloc bay, Philippines. Toxicon.

[35] Llewellyn L., Negri A., Robertson A. (2006). Paralytic shellfish toxins in tropical oceans. Toxin Rev..

[36] Azanza R.V., Miranda L.N. (2001). Phytoplankton composition and Pyrodinium bahamense toxic blooms in Manila Bay, Philippines. J. Shellfish Res..

[37] Azanza R.V., Taylor F.J.R. (2001). Are Pyrodinium blooms in the Southeast Asian region recurring and spreading? A view at the end of the millennium. AMBIO.

[38] Gacutan R.Q., Tabbu M.Y., Aujero E.J., Icatlo F. (1985). Paralytic shellfish poisoning due to pyrodinium-bahamense var compressa in Mati, Davao-Oriental, Philippines. Mar. Biol..

[39] Bodager D. (2002). Outbreak of saxitoxin illness following consumption of Florida pufferfish. Fl J. Environ. Health.

[40] Brandenburg K.M., Wohlrab S., John U., Kremp A., Jerney J., Krock B., Van de Waal D.B. (2018). Intraspecific trait variation and trade-offs within and across populations of a toxic dinoflagellate. Ecol. Lett..

[41] Murray S.A., Mihali T.K., Neilan B.A. (2011). Extraordinary conservation, gene loss, and positive selection in the evolution of an ancient neurotoxin. Mol. Biol. Evol..

[42] Zhang Y., Zhang S.F., Lin L., Wang D.Z. (2014). Comparative transcriptome analysis of a toxin-producing dinoflagellate Alexandrium catenella and its non-toxic mutant. Mar. Drugs.

[43] John U., Beszteri B., Derelle E., Van de Peer Y., Read B., Moreau H., Cembella A.D. (2008). Novel insights into evolution of protistan polyketide synthases through phylogenomic analysis. Protist.

[44] Eichholz K., Beszteri B., John U. (2012). Putative monofunctional type I polyketide synthase units: A dinoflagellate-specific feature?. PLoS ONE.

[45] Nosenko T., Bhattacharya D. (2007). Horizontal gene transfer in chromalveolates. BMC Evol. Biol..

[46] Wisecaver J.H., Brosnahan M.L., Hackett J.D. (2013). Horizontal gene transfer is a significant driver of gene innovation in dinoflagellates. Genome Biol. Evol..

[47] Altschul S.F., Gish W., Miller W., Myers E.W., Lipman D.J. (1990). Basic local aligment search tool. J. Mol. Biol..

[48] Cusick K.D., Wilhelm S.W., Hargraves P.E., Sayler G.S. (2016). Single-cell PCR of the luciferase conserved catalytic domain reveals a unique cluster in the toxic bioluminescent dinoflagellate Pyrodinium bahamense. Aquat. Biol..

[49] Tomas C.R. (1997). Identifying Marine Phytoplankton.

[50] Zhang Z., Schwartz S., Wagner L., Miller W. (2000). A greedy algorithm for aligning DNA sequences. J. Comput. Biol..

[51] Phlips E.J., Badylak S., Grosskopf T. (2002). Factors affecting the abundance of phytoplankton in a restricted subtropical lagoon, the Indian River Lagoon, Florida, USA. Estuar. Coast. Shelf Sci..

[52] Badylak S., Phlips E.J. (2004). Spatial and temporal patterns of phytoplankton composition in a subtropical coastal lagoon, the Indian River Lagoon, Florida, USA. J. Plankton Res..

[53] Phlips E.J., Badylak S., Christman M.C., Lasi M.A. (2010). Climatic trends and temporal patterns of phytoplankton composition, abundance and succession in the Indian River Lagoon, Florida, USA. Estuar. Coast..

[54] Phlips E.J., Badylak S., Bledsoe E., Cichra M. (2006). Factors affecting the distribution of Pyrodinium bahamense var. bahamense in coastal waters of Florida. Mar. Ecol. Prog. Ser..

[55] Grasso S., Albrecht M., Bras M.M. (2016). Seasonal abundance of Pyrodinium bahamense (order Peridiniales, family Gonyaulacaceae) in Mosquito Bay, Vieques, Puerto Rico. J. Coast Life Med..

[56] Steidinger K.A., Tester L.S., Taylor F.J.R. (1980). A redescription of Pyrodinium bahamense var. compressa (Bohm) stat. nov. from Pacific red tides. Phycologia.

[57] Mertens K.N., Wolny J., Carbonell-Moore C., Bogus K., Ellegaard M., Limoges A., de Vernal A., Gurdebeke P., Omura T., Al-Muftah A. (2015). Taxonomic re-examination of the toxic armored dinoflagellate Pyrodinium bahamense Plate 1906: Can morphology or LSU sequencing separate P. bahamense var. compressum from var. bahamense?. Harmful Algae.

[58] Liu L., Wilson T., Hastings J.W. (2004). Molecular evolution of dinoflagellate luciferases, enzymes with three catalytic domains in a single polypeptide. Proc. Natl. Acad. Sci. USA.

[59] Okamoto O.K., Liu L., Robertson D.L., Hastings J.W. (2001). Members of a dinoflagellate luciferase gene family differ in synonymous substitution rates. Biochemistry.

