# Supplementary material for: sxtA4+ and sxtA4- Genotypes Occur Together within Natural Pyrodinium bahamense Sub-Populations from the Western Atlantic
Source: Microorganisms. 2021 May 23;9(6):1128. doi: 10.3390/microorganisms9061128 (PMC8224543; doi:10.3390/microorganisms9061128)
Supplement: Supplementary file 1 [file microorganisms-09-01128-s001.zip › microorganisms-1187865-supplementary.pdf]

Methods for Additional Screening of Samples Yielding Only 18S rRNA Gene Amplicon in Initial Multiplex:

Samples which yielded only an 18S rRNA gene amplicon in the initial multiplex were further screened with additional PCRs using the sxtA4 primer pair 166F/680R. 166F targets a conserved region within sxtA4 of both *Pyrodinium* and *Alexandrium* spp (see sequence alignments under "sxtA4 Primer Variability" below). As the initial multiplex PCR was performed in the same tube that contained the single cell, all genomic DNA was still present in the tube for subsequent PCRs. These additional PCRs utilized as template the original multiplex PCR prepared as follows: 2 ul of a 1:5 dilution of the original multiplex; 20 ul undiluted; 2ul undiluted;14 ul cleaned PCR following the genomic DNA clean-up method of the GeneClean Turbo Clean-Up kit (MoBio). The PCRs that utilized 2 ul of a 1:5 dilution of the original multiplex PCR as template were the equivalent of the nested PCR used to screen the multiplex PCRs that yielded bands indicative of 18S rRNA gene and *sxtA4*. PCRs were conducted in 25 ul volumes using the conditions and thermocycling conditions described for the nested PCRs performed with the 166F/680R primers on samples that yielded an *sxtA4* amplicon in the initial multiplex. PCRs were also performed on the initial multiplex samples that yielded only the 18S rRNA gene amplicon using the F1/680R primers alone using 2 ul undiluted and 2 ul of a 1:5 dilution of the original PCR as template. Reagents and thermocycling conditions were the same as those used for the 166F/680R nested PCR.

Table S1. STX congeners detected in toxic *P. bahamense* lab isolate from the Indian River Lagoon.

[illegible]

| ESF ID # | Culture | Sample Name | Total PST in µg STX eq./L culture by HPLC-FL) | Congeners Present |
|---|---|---|---|---|
| 19-468 | Pyrodinium bahamense | 1 = No Copper, High Light, t= 1 day, Day Phase | 41.28 | GTX 5, STX |
| 19-469 | Pyrodinium bahamense | 2 = 1 uM Cu, High Light, t= 1 day, Day Phase | 38.44 | GTX 5, STX |
| 19-470 | Pyrodinium bahamense | 3 = 10 uM Cu, High Light, t = 1 day, Day Phase | 69.34 | GTX 5, STX |
| 19-471 | Pyrodinium bahamense | 4 = No Copper, Low Light, t = 1 day, Day Phase | 35.84 | GTX 5, STX |
| 19-472 | Pyrodinium bahamense | 5 = 1 uM Copper, Low Light, t = 1 day, Day Phase | 32.47 | GTX 5, STX |
| 19-473 | Pyrodinium bahamense | 6 = 10 uM Copper, Low Light, t =1 day, Day Phase | 38.74 | GTX 5, STX |
| 19-474 | Pyrodinium bahamense | 7 = No Copper, High Light, t = 5 day, Dark Phase | 130.66 | GTX 5, STX |
| 19-475 | Pyrodinium bahamense | 8 = 10 uM Cu, High Light, t = 5 day, Dark Phase | 93.39 | GTX 5, STX |
| 19-476 | Pyrodinium bahamense | 9 = 1uM Cu, High Light, t = 5 day, Dark Phase | 207.87 | GTX 5, STX |
| 19-477 | Pyrodinium bahamense | 10 = No Copper, Low Light, t = 5 day, Dark Phase | 104.30 | GTX 5, STX |
| 19-478 | Pyrodinium bahamense | 11 = 1 uM Cu, Low Light, t = 5 day, Dark Phase | 106.19 | GTX 5, STX |
| 19-479 | Pyrodinium bahamense | 12 = 10 uM Cu, Low Light, t = 5 day, Dark Phase | 174.68 | GTX 5, STX |

Methods for Toxin Analysis: Toxin analysis was performed at the State University of New York College of Environmental Science and Forestry. The cellular material was combined with 250 µL of 1% acetic acid in water and freeze-thawed three times to extract PSTs. Extracts were clarified by centrifugation at 16,000×g and the supernatant analyzed in triplicate using HPLC-Fluorescence with post-column chemical oxidation to determine PSTs. All toxins were quantified in STX equivalents, using STX standards acquired from Canada's National Research Council, as well as secondary in-house standards. The method detection limit was 0.48-0.58 µg STX eq/L, which is comparable or better than other established methods. Two PST congeners were evident in the samples. Identity was determined by matching retention times of standards, including GTX 1, 2, 3, 4, 5, dcGTX 2,3, C1/C2, STX, dcSTX, NEO, and LWTX-1,2,3,5,6.

Table S2. Percent Identity between two representative sequences from clone sequences of individual P. bahamense cells and published *P. bahamense* sequences.

| | LW24_7 (-15bp)** | LW24_1 | PR46_6 (-15)** | PR46_9 |
|---|---|---|---|---|
| MN431957.1* | 95% (358bp) | 98% (358bp) | 95% (358 bp) | 99% (358bp) |
| GBXF01000001.1 TSA | 96% (502bp) | 98% (517bp) | 96% (502bp) | 99% (517bp) |

*MN431957.1 is a 358 bp sequence in GenBank.

** indicates 15 bp deletion in sequence

sxtA4 Primer Variability: The following representative sequences illustrate the sequence differences in the sxtA4F1 and sxtA4166F primers between *Pyrodinium* and *Alexandrium* spp. *Pyrodinium* sequences include clone sequences from both genomic DNA and cDNA from the toxic lab isolate amplified with the 007F/sxtA4680R primers. Yellow highlight indicates sxtA4F1 primer binding region; base differences between *Pyrodinium* and *Alexandrium* are indicated in bold, with *Pyrodinium* sequence in brackets. Green highlight indicates sxtA4166F primer region.

>IRL_sxtADNA 1_007/680R

ATGCTCAACATGGGAGTCATCCCGAGCCTCGTGGGCGAGAGCGGGTTCCTCCTCCTGGACAT
AAACGCCCATGACTGCGTGCAGACGGCCGCCAGGCTCTGCAAGAAGGGCGCCACCGTCGTG
CGCCTGAAGCACAACGACATGAAGCAGCTCGAGCGCGTGGTCTCGTCGATCCCGGAGGCGG
CCGACATCACCTACGTGTGCGACGGCGTTTACTCCACGGACGGAGAGCTCGCCGACTTGCCC
GCCATATGTGCTTGTTTGAGGCCACGCGGGGCCAAGATACTCGTAGACGACTCACATGGCTG
CGGCGTTCTTGGCCGCAACCCCGACTCGGAACAACCCCTCGGATATGGTGGCGGCGGCGTCG
TCAAGTACTTCGGGCTGGACTATGCGGAGAACAACATCATCTACGCCGGCCAGCTGAGCAA
GGCGTTCAATTCGCCCGGCGGATTCGTCGGCTGCGCACGCGAGACCGACGAGAAGTTCGGC
ATTCTGAACTTGGCCAAGAACTCGAACACGCTCGTGTTCACAGGCCCGATCTGTACTGCCGG
CCTGTCGAGTGCGAAGACGACCCTCGACCTCAACGCCGCTGAGGGGGACCTTCAGCGCAAG
CGGCTTCTGGAGGCGACCCTCGGATTCTGCGAGGGGCTCAAGGCACTCGGGTGCCCCCACAC
CTACCACGGATTCCCCGTCGTCAACATCTACTGGACCCCGGTCCAGGTGTGCGCAGAGGTGT
ACAGGGAGCTGATGAGCGCGAGGCAGGGCGCGTTCCAGCGGGGCGTCATCACGACCCCTAT
GTGGTACCCCATCTAG

>IRL_sxtA4DNA_2_007/680R

ATGCTCAACATGGGAGTCATCCCGAGCCTCGTGGGCGAGAGCGGGTTCCTCCTCCTGGACAT
AAACGCCCATGACTGCGTGCAGACGGCCGCCAGGCTCTGCAAGAAGGGCGCCACCGTCGTG
CGCCTGAAGCACAACGACATGAAGCAGCTCGAGCGCGTGGTCTCGTCGATCCCGGAGGCGG
CCGACATCACCTACGTGTGCGACGGCGTTTACTCCACGGACGGAGAGCTCGCCGACTTGCCC
GCCATATGTGCTTGTTTGAGGCCACGCGGGGCCAAGATACTCGTAGACGACTCACATGGCTG
CGGCGTTCTTGGCCGCAACCCCGACTCGGAACAACCCCTCGGATATGGTGGCGGCGGCGTCG
TCAAGTACTTCGGGCTGGACTATGCGGAGAACAACATCATCTACGCCGGCCAGCTGAGCAA
GGCGTTCAATTCGCCCGGCGGATTCGTCGGCTGCGCACGCGAGACCGACGAGAAGTTCGGC
ATTCTGAACTTGGCCAAGAACTCGAACACGCTCGTGTTCACAGGCCCGATCTGTACTGCCGG
CCTGTCGAGTGCGAAGACGACCCTCGACCTCAACGCCGCTGAGGGGGACCTTCAGCGCAAG
CGGCTTCTGGAGGCGACCCTCGGATTCTGCGAGGGGCTCAAGGCACTCGGGTGCCCCCACAC
CTACCACGGATTCCCCATCGTCAACATCTACTGGACCCCGGTCCAGGTGTGCGCAGAGGTGT
ACAGGGAGCTGATGAGCGCGAGGCAGGGCGCGTTCCAGCGGGGCGTCATCACGACCCCTAT
GTGGTACCCCATCTAG

>IRL_sxtA4DNA_3_007/680R

ATGCTCAACATGGGAGTCATCCCGAGCCTCGTGGGCGAGAGCGGGTTCCTCCTCCTGGACAT
AAACGCCCATGACTGCGTGCAGACGGCCGCCAGGCTCTGCAAGAAGGGCGCCACCGTCGTG
CGCNTGAAGCACAACGACATGAAGCAGCTCGAGCGCGTGGTCTCGTCGATCCCGGAGGCGG
CCGACATCACCTACGTGTGCGACGGCGTTTACTCCACGGATGGAGAGCTCGCCGACTTGCCC
GCCATATGTGCTTGTTTGAGGCCACGCGGGGCCAAGATACTCGTAGACGACTCACATGGCTG
CGGCGTTCTTGGCCGCAACCCCGACTCGGAACAACCCCTCGGATATGGTGGCGGCGGCGTCG
TCAAGTACTTCGGGCTGGACTATGCGGAGAACAACATCATCTACGCCGGCCAGCTGAGCAA
GGCGTTCAATTCGCCCGGCGGATTCGTCGGCTGCGCACGCGAGACCGACGAGAAGTTCGGC
ATTCTGAACTTGGCCAAGAACTCGAACACGCTCGTGTTCGCAGGCCCGATCTGTACTGCCGG
CCTGTCGAGTGCGAAGACGACCCTCGACCTCAACGCCGCTGAGGGGGACCTTCAGCGCAAG
CGGCTTCTGGAGGCGACCCTCCGATTCTGCGAGGGGCTCAAGGCACTCGGGTGCCCCCACAC
CTACCACGGATTCCCCATCGTCAACATCTACTGGACCCCGGTCCAGGTGTGCGCAGAGGTGT
ACAGGGAGCTGATGAGCGCGAGGCAGGGCGCGTTCCAGCGGGGCGTCATCACGACCCCTAT
GTGGTACCCCATCTAG

>IRL_sxtA4DNA_4_007/680R

ATGCTCAACATGGGAGTCATCCCGAGCCTCGTGGGCGAGAGCGGGTTCCTCCTCCTGGACAT
AAACGCCCATGACTGCGTGCAGACGGCCGCCAGGCTCTGCAAGAAGGGCGCCACCGTCGTG
CGCCTGAAGCACAACGACATGAAGCAGCTCGAGCGCGTGGTCTCGTCGATCCCGGAGGGGG
CCGACATCACCTACGTGTGCGACCGCGTTTATTCCACGGACGGAGAGCTTGCCGACTTGCCC
GCCATATGTGCTTGTTTGAGGCCACGCGGGGCCAAGATACTCGTAGACGACTCGCATGGCTG
CGGCGTTCTTGGCCGCAACCCCGACTCGGAACGACCCCTCGGATATGGTGGCGGCGGCGTCG
TCAAGTACTTCGGGCTGGACTACGCGGAGAATAACATTTACGCCGGGCAACTTAGCAAGGC
GTTCAATTCGCCCGGCGGATTCGTCGGCTGCGCATGCGAGACCAACGAGTAGCTCGGCATTC
TAAACTTGGCCAAGAACTCGAACACGCTCGTGTTCACAGGCCCGATCTGTACTGCCGGCCTG
TCGAGTGCGAAGACGACCCTCGACCTCAACGCCGCTGAGGGGGGCCTTCAGCGCAAGCGGC
TTCTGGAGACGACCTCGGATTCTGCGAGGGGCTCAAGGCACTCGGGTGCCCCACACCTACCA
CGGATTCCCCATCGTCAACATCTACTGGACCCCGGTCGAGACGTGCGCAGAGGTGCACAGG
GAGCTGATGAGCGCGAGACAGGGCGCGTTCCAGCGGGGCGTCATCACGACCCCTATGTGGT
ACCCCATCTAG

>IRL_sxtA4_DNA_5_007/680R

ATGCTCAACATGGGAGTCATCCCGAGCCTCGTGGGCGAGAGCGGGTTCCTCCTCCTGGACAT
AAACGCCCATGACTGCGTGCAGACGGCCGCCAGGCTCTGCAAGAAGGGCGCCACCGTCGTG
CGCCTGAAGCACAACGACATGAAGCAGCTCGAGCGCGTGGTCTCGTCGATCCCGGAGGCGG
CCGACATCACCTACGTGTGCGACGGCGTTTACTCCACGGACGGAGAGCTCGCCGACTTGCCC
GCCATATGTGCTTGTTTGAGGCCACGCGGGGCCAAGATACTCGTAGACGACTCACATGGCTG
CGGCGTTCTTGGCCGCAACCCCGACTCGGAACAACCCCTCGGATATGGTGGCGGCGGCGTCG
TCAAGTACTTCGGGCTGGACTATGCGGAGAACAACATCATCTACGCCGGCCAGCTGAGCAA
GGCGTTCAATTCGCCCGGCGGATTCGTCGGCTGCGCACGCGAGACCGACGAGAAGTTCGGC
ATTCTGAACTTGGCCAAGAACTCGAACACGCTCGTGTTCACAGGCCCGATCTGTACTGCCGG
CCTGTCGAGTGCGAAGACGACCCTCGACCTCACCGCCGCTGAGGGGGACCTTCAGCGCAAG
CGGCTTCTGGAGGCGACCCTCGGATTCTGCGAGGGGCTCAAGGCACTCGGGTGCCCCCACAC
CTACCACGGATTCCCCATCGTCAACATCTACTGGACCCCGGTCCAGGTGTGCGCAGAGGTGT

ACAGGGAGCTGATGAGCGCGAGGCAGGGCGCGTTCCAGCGGGGCGTCATCACGACCCCTAT
GTGGTACCCCATCTAG

>IRL_ sxtA4cDNA_1_007/680R

ATGCTCAACATGGGAGTCATCCCGAGCCTCGTGGGCGAGAGCGGGTTCCTCCTCCTGGACAT
AAACGCCCATGACTGCGTGCAGACGGCCGCCAGGCTCTGCAAGAAGGGCGCCACCGTCGTG
CGCCTGAAGCACAACGACATGAAGCAGCTCGAGCGCGTGGTCTCGTCGATCCCGGAGGCGG
CCGACATCACCTACGTGTGCGACGGCGTTTACTCCACGGACGGAGAGCTCGCCGACTTGCCC
GCCATATGTGCTTGTTTGAGGCCACGCGGGGCCAAGATACTCGTAGACGACTCACATGGCTG
CGGCGTTCTTGGCCGCAACCCCGACTCGGAACAACCCCTCGGATATGGTGGCGGCGGCGTCG
TCAAGTACTTTGGGCTGGACTACGCGGAGAACAACATCATCTACGCCGGCCAGCTGAGCAA
GGCGTTCAATTCGCCCGGCGGATTCGTCGGCTGCGCACGCGAGACCGACGAGAAGTTCGGC
ATTCTGAACTTGGCCAAGAACTCGAACACGCTCGTGTTCACAGGCCCGATCTGTACTGCCGG
CCTGTCGAGTGCGAAGACGACCCTCGACCTCAACGCCGCTGAGGGGGACCTTCAGCGCAAG
CGGCTTCTGGAGGCGACCCTCGGATTCTGCGAGGGGCTCAAGGCACTCGGGTGCCCCCACAC
CTACCACGGATTCCCCATCGTCAACATCTACTGGACCCCGGTCCAGGTGTGCGCAGAGGTGT
ACAGGGAGCTGATGAGCGCGAGGCAGGGCGCGTTCCAGCGGGGCGTCATCACGACCCCTAT
GTGGTACCCCATCTAG

>IRL_ sxtA4cDNA_2_007/680R

ATGCTCAACATGGGAGTCATCCCGAGCCTCGTGGGCGAGAGCGGGTTCCTCCTCCTGGACAT
AAACGCCCATGACTGCGTGCAGACGGCCGCCAGGCTCTGCAAGAAGGGCGCCACCGTCATG
CGCCTGAAGCACAACGACATGAAGCAGCTCGAGCGCGTGGTCTCGTCGATCCCGGAGGCGG
CCGACATCACCTACGTGTGCGACGGCGTTTACTCCACGGACGGAGAGCTCGCCGACTTGCCC
GCCATATGTGCTTGTTTGAGGCCACGCGGGGCCAAGATACTCGTAGACGACTCACATGGCTG
CGGCGTTCTTGGCCGCAACCCCGACTCGGAACAACCCCTCGGATATGGTGGCGGCGGCGTCG
TCAAGTACTTTGGGCTGGACTACGCGGAGAACAACATCATCTACGCCGGCCAGCTGAGCAA
GGCGTTCAATTCGCCCGGCGGATTCGTCGGCTGCGCACGCGAGACCGACGAGAAGTTCGGC
ATTCTGAACTTGGCCAAGAACTCGAACACGCTCGTGTTCACAGGCCCGATCTGTACTGCCGG
CCTGTCGAGTGCGAAGACGACCCTCGACCTCAACGCCGCTGAGGGGGACCTTCAGCGCAAG
CGGCTTCTGGAGGCGACCCTCGGATTCTGCGAGGGGCTCAAGGCACTCGGGTGCCCCCACAC
CTACCACGGATTCCCCATCGTCAACATCTACTGGACCCCGGTCCAGGTGTGCGCAGAGGTGT
ACAGGGAGCTGATGAGCGCGAGGCAGGGCGCGTTCCAGCGGGGCGTCATCACGACCCCTAT
GTGGTACCCCATCTAG

>GBXF01000001.1 TSA: Pyrodinium bahamense var. compressum TSA:Pbc_sxtA4

AACGACATGAAGCAGCTCGAGCGCGTGGTCTCGTCGATCCCGGAGGGGGCCGACATCACCT
ACGTGTGCGACGGCGTTTACTCCACGGACGGAGAGCTCGCCGACTTGCCCGCCATATGTGCT
TGTTTGAGGCCACGCGGGGCCAAGATACTCGTAGACGACTCACATGGCTGCGGCGTTCTTGG
CCGCAACCCCGACTCGGAACAACCCCTCGGATATGGTGGCGGCGGCGTCGTCAAGTACTTCG
GGCTGGACTACGCGGAGAACAACATCATCTACGCCGGCCAGCTGAGCAAGGCGTTCAATTC
GCCCGGCGGATTCGTCGGCTGCGCACGCGAGACCGACGAGAAGTTCGGCATTCTGAACTTG
GCCAAGAACTCGAACACGCTCGTGTTCACAGGCCCGATCTGTACTGCCGGCCTGTCGAGTGC
GAAGACGACCCTCGACCTCAACGCCGCTGAGGGGGACCTTCAGCGCAAGCGGCTTCTGGAG
GCGACCCTCGGATTCTGCGAGGGGCTCAAGGCACTCGAGTGCCCCCACACCTACCACGGATT
CCCCAT

CGTCAACATCTACTGGACCCCGGTCCAGGTGTGCGCAGAGGTGTACAGGGAGCTGATGAGC
GCGAGGCAGGGCGCGTTCCAGCGGGGCGTCATCACGACCCCTATGTGGTACCCCATCTAG

>JF343394.1 Alexandrium fundyense strain CCMP1719 clone L38

GCTCAACATGGGAGTCATCCCGAGCCTCGTGGGCGAGAGCGGGTTCCTCCTCCTGGACATAA
ACGCCCACGACTGCGTGCAGACGGCCGCCAGGCTCTGCAAGAAGGGCGCCACCGTGGTGCG
CCTGAAGCACAACGAC**G[A]C[T]GG[A]**AGCAGCTCGAGCACATGCTCTCGTCGATCCCGCAG
GGGGCCGACATCACCTACGTGTGCGACGGCGTGTACTCCACGGACGGAGAGCTCGCCGACT
TGCCCGCCATATGTGCTTGTTTGAGGCCGCGCGGGGCCAAGATACTCGTAGACGACTCGCAT
GGCTGCGGCGTTCTTGGCCGCAACCCCGACTCGGAGCAACCCTTCGGGTATGGTGGCGGCGG
CGTCGTCGAGTACTTCGGGCTGGACTACGCGGAGAACAACATCATCTACGCCGGGCAGCTG
AGCAAGGCGTTCAATTCGCCCGGCGGATTCGTCGGCTGTGCGCGCGAGACCGACGAGAAGT
TCGGCATTCTGAACTTGGCCAAGAACTCAAACACACTCGTGTTCACAGGGCCGATCTGTACT
GCCGGCCTGTCGAGTGCGATGACGACCCTCGACCTCAACGCCGCCGAGGGGGACCTTCAGC
GCAAGCGGCTTCTGGCGGCGACCCTCGAATTCTGCGAGGGGCTCAAGGCGCTCGGGTGCCCC
CACACCTACCACGGGTTCCCCATCGTCAACATCTACTGGACCCCGGTCGAGGTGTGCGCAGA
GGTGTACAGGGAGCTGATGAGCGCGAGGCAGGGCGCGTTCCAGCTGGGCGTCGTCACGACC
CCCATGTGGCACCCCATCGCCCCAAAGGGCCACGAGATACTGCGCTTCCAGTTCACGTCGCT
CCACGACGAGGCCGCCGTGCGCCACATCCTCGTGATCCTCGAGGACCTGATCAAGCGCTACC
CGCCCTCCGCCGTGCCGCCGCGCATCTGATCGGCTGCCCGAGCCGCAGGACCAGCGCCGCTC
ATCCCAGGGGTTGTTTAAGGGATTGTTGAGTCTTTTCAATCTAGTCAGCGTGTTTTTAATGTG
CAAGCAGCAAGGGTCAGGCGGATTCTGGGCTTGTACACCAAGGGCCAGGCAGGTTTTGGCT
GCCGCCGTTTTGATCCTGCTGTGTTGTCGTAGCGTGCAAGCAGCAAGGGTCAGGCGGAGTCT
GGGCTTGTACACCAAGGGCCAGGCAGGTTTTGGCTGCCGCCGTTTTGATCCTGCTGTGTTGT
GGTAGCATGCAAGCAGCAAGGGTCAGGCGGATTTTGTGGCTTTGCTGATCCTGATCCAAGGT
ATGACCAGCCATGGCGCATCATCGCGTCTAATGTAGCGCCTGTCCATGTCTGCCATCTTAGCT
GGTCACTATTTGCATCAACACTCGCAAGGTACGCGTCTGCTACGCACAGGTGACAATTGACA
TTGTGGATCGAGCCACGGAAGGGAGAAAAAAAAAAAAAA

>JF343393.1 Alexandrium fundyense strain CCMP1719 clone L37

TGCTCAACATGGGAGTCATCCCGAGCCTCGTGGGCGAGAGCGGGTTCCTCCTCCTGGACATA
AACGCCCACGACTGCGTGCAGACGGCCGCCAGGCTCTGCAAGAAGGGCGCCACCGTGGTGC
GCCTGAGGCACAACGAC**G[A]C[T]GG[A]**AGCAGCTCGAGCACATGCTCTCGTCGATCCCGCA
GGGGGCCGACATCACCTACGTGTGCGACGGCGTGTACTCCACGGACGGAGAGCTCGCCGAC
TTGCCCGCCATATGTGCTTGTCTGAGGCCGCGCGGGGCCAAGATACTCGTAGACGACTCGCA
TGG**T[C]**TGCGGCGTTCTTGGCCGCAACCCCGACTCGGAGCAACCCTTCGGGTATGGTGGCGG
CGGCGTCGTCGAGTACTTCGGGCTGGACTACGCGGAGAACAACATCATCTACGCCGGGCAG
CTGAGCAAGGCGTTCAATTCGCCCGGCGGATTCGTCGGCTGTGCGCGCGAGACCGACGAGA
AGTTCGACATTCTGAACTTGGCCAAGAACTCGAACACACTCGTGTTCACAGGGCCGATCTGT
ACTGCCGGCCTGTCGAGTGCGATGACGACCCTCGACCTCAACGCCGCCGAGGGGGACCTTCA
GCGCAAGCGGCTTCTGGCGGCGACCCCCGAATTCTGTGAGGGGCTCAAGGCGCTCGGGTGC
CCCCACACCTACCACGGGTTCCCCATCGTCAACATCTACTGGACCCCGGTCGAGGTGTGCGC
AGAGGTGTACAGCGAGCTGATGAGCGCGAGGCAGGGCGCGTTCCAGCTGGGCGTCGTCACG
ACCCCCATGTGGCACCCCATCGCTCCGAAGGGCCACGAGATGCTGCGCTTCCAGTTCACGTC
GCTCCACGACGAGGCCGCCGTGCGCCACATCCTCGTGATCCTCGAGGACCTGATCAAGCGCT

ACCCGCCCTCCGCCGTGCCGCCGCGCATCTGATCGGCCGCCCGAGCCGCAGGACCAGCGCCG
CTCATCCCAGGGGTTGTTTAAGGGATTGTTTAATCTTCTCAATCTAGTCAGCGTGTTTTTAAT
GTGCAAGCAGCAAGGGTCAAGCGGATTCTGGGCTTGTACACCAAGGGCCAGGCAGGTTTTG
GCTGCCGCCATTTTGATCCTGCTGTGTTGTCGTAGCGTGCAAGCAGCAAGGGCCAGGCGGAG
TCTGTGCTTGTACACCAAGGGCCAGGCAGGTTTTGGCTGCCGCCGTTTTTATCCGGCTGTGTT
GTGGTAGCGTGCAAGCAGCAAGGGTCAGGCGGATTTGGGGCTTTGCTGATCCTGATCCAAG
GTAAAAAAAAAAAAAAAAAAAAAAAAAA

>JF343351.1 Alexandrium minutum strain CCMP1888 clone cl07/03

TGCTCAACATGGGAGTCATCCCGAGCCTGGCGGGCGGGAGCGGGTTCCTCCTCCTGGACATA
AACGCCCACGACTGCGTGCAGACGGCAGCCAGGCTCTGCAAGAAGGGAGTCACCGTCGTGC
GCCTGAAGCACAACGACA**C[T]**G**G[A]**AGCAGCTCGAGCGCGTGCTCTCGTCGATCCCGGAGG
GGGCCGACATCACCTACGTGTGCGACGGCGTCTACTCCACGGACGGAGAGCTCGCCGACCT
GCCCGCCATATGTGCTTGTTTGAGGCCGCGCGGGGCCAAGATACTCGTGGACGACTCG**CAC[**
**T]**GGTTGCGGCGTTCTTGGCCGCAACCCCGACTCGGAGCAACCCTTCGGATATGGCGGCGGC
GGCGTCGTCAAGTACTTCGGGCTGGACTACGCGGAGAACAACATCATCTACGCCGGGCAGC
TGAGCAAGGCGTTCAATTCGCCCGGCGGATTCGTGGGCTGCGCACGCGAGACCGACGAGAA
GTTCGGCATTCTGAACTTGGCCAAGAACTCGAACACGCTCGTGTTCACAGGGCCGATCTGTA
CTGCCGGCCTGTCGAGTGCGAAGACGACCCTCGACCTCAACTTTGCCGAGGGGGACCGTCAG
CGCAAGCGGCTTCTTGAGGCGACCCTGGAATTTTGCGAGGGGCTCAAGGCGCTCGGGTGCCC
CCACACCTACCACGGGTTCCCCATCGTCAACATCTACTGGACCC

Representative 18S rRNA gene sequences for alignment as referred to in the Discussion. “IRL” refers to Indian River Lagoon sequences from this study.
>IRL_DB
CAGGGCATCCATGTCTTGTAATTGGAATGAATGGAAATTAAACCTCTTTGTAAGTATCGATT
GGAGGGCAAGTCTGGTGCCAGCAGCCGCGGTAATTCCAGCTCCAATAGCGTATATTAAAGTT
GTTGCGGTTAAAAAGCTCGTAGTTGGATTTCTGCTAAGGGTGGCTGGTCCGCCCTCTGGGTG
AGTATCTGGCACAACCTGAGCATCTTTCTGGAGAACGTAACTGCACTTGACTGTGTGGTGCG
GTATCGAGAACTTTTACTTTGAGGAAATCAGAGTGTTTCAAGCAGGTGTTTGCCTTGAATAC
ATTAGCATGGAATAATAACATAGGACTTTGGTTCTATTTTGTTGGTTTCTAGAACGAAAGTA
ATGATTAATAGGGATAGTTGGGGGCATTCGTATTTAATTGTCAGAGGTGAAATTCTTGGATT
TGTTGAAGACGGACTACTGCGAAAGCATTTGCCAAGGATGTTTTCATTGATCAAGAACGAAA
GTTAGGGGATCGAAGACGATCAGATACCGTCCTAGTCTTAACCATAAACCATGCCAACTAGA
GATTGGAGGCCGTTATTTGTACGATTCCTTCAGCACCTTATGAGAAATCGAAGTCTTTGGGTT
CCGGGGGGAGTATGGTCGCAAGGCTGAAACTTAAAGGAATTGACGGAAGGGCACCACCAGG
AGTGGAGCCTGCGGCTTAATTTGACTCAACACGGGGAAACTTACCAGGTCCAGACATAATTA
GGATTGACAGATTGATAGCTCTTTCTTGATTCTATGGGTGGTGGTGCATGGCCGTTCTTAGTT
GGTGGAGTGATTTGTCTGGTTAATTCCGTTAACGAACGAGACCTTAACCTGCTAAATAGTTA
CACATAATTCCGGTTATGTGGGCAACTTCTTA
>IRL_520
CAGGGCATCCATGTCTTGTAATTGGAATGAATGGAAATTAAACCTCTTTGTAAGTATCGATT
GGAGGGCAAGTCTGGTGCCAGCAGCCGCGGTAATTCCAGCTCCAATAGCGTATATTAAAGTT
GTTGCGGTTAAAAAGCTCGTAGTTGGATTTCTGCTAAGGGTGGCTGGTCCGCCCTCTGGGTG
AGTATCTGGCACAACCTGAGCATCTTTCTGGAGAACGTAACTGCACTTGACTGTGTGGTGCG
GTATCGAGAACTTTTACTTTGAGGAAATCAGAGTGTTTCAAGCAGGTGTTTGCCTTGAATAC

ATTAGCATGGAATAATAACATAGGACTTTGGTTCTATTTTGTTGGTTTCTAGAACGAAAGTA
ATGATTAATAGGGATAGTTGGGGGCATTCGTATTTAATTGTCAGAGGTGAAATTCTTGGATT
TGTTGAAGACGGACTACTGCGAAAGCATTTGCCAAGGATGTTTTCATTGATCAAGAACGAAA
GTTAGGGGATCGAAGACGATCAGATACCGTCCTAGTCTTAACCATAAACCATGCCAACTAGA
GATTGGAGGCCGTTATTTGTACGATTCCTTCAGCACCTTATGAGAAATCGAAGTCTTTGGGTT
CCGGGGGGAGTATGGTCGCAAGGCTGAAACTTAAAGGAATTGACGGAAGGGCACCACCAGG
AGTGGAGCCTGCGGCTTAATTTGACTCAACACGGGGAAACTTACCAGGTCCAGACATAATTA
GGATTGACAGATTGATAGCTCTTTCTTGATTCTATGGGTGGTGGTGCATGGCCGTTCTTAGTT
GGTGGAGTGATTTGTCTGGTTAATTCCGTTAACGAACGAGACCTTAACCTGCTAAATAGTTA
CACATAATTCCGGTTATGTGGGCAACTTCTTA
>DQ500123.1 Pyrodinium bahamense var. compressum PYRO 18S
CAGGGCATCCATGTCTTGTAATTGGAATGAATGGAAATTAAACCTCTTTGTAAGTATCGATT
GGAGGGCAAGTCTGGTGCCAGCAGCCGCGGTAATTCCAGCTCCAATAGCGTATATTAAAGTT
GTTGCGGTTAAAAAGCTCGTAGTTGGATTTCTGCTAAGGGTGGCTGGTCCGCCCTCTGGGTG
AGTATCTGGCACAACCTGAGCATCTTTCTGGAGAACGTAACTGCACTTGACTGTGTGGTGCG
GTATCGAGAACTTTTACTTTGAGGAAATCAGAGTGTTTCAAGCAGGTGTTTGCCTTGAATAC
ATTAGCATGGAATAATAACATAGGACTTTGGTTCTATTTTGTTGGTTTCTAGAACGAAAGTA
ATGATTAATAGGGATAGTTGGGGGCATTCGTATTTAATTGTCAGAGGTGAAATTCTTGGATT
TGTTGAAGACGGACTACTGCGAAAGCATTTGCCAAGGATGTTTTCATTGATCAAGAACGAAA
GTTAGGGGATCGAAGACGATCAGATACCGTCCTAGTCTTAACCATAAACCATGCCAACTAGA
GATTGGAGGCCGTTATTTGTACGATTCCTTCAGCACCT-
ATGAGAAATCGAAGTCTTTGGGTTCCGGGGGGAGTATGGTCGCAAGGCTGAAACTTAAAGG
AATTGTCGGAAGGGCACCACCAGGAGTGGAGCCTGCGGCTTAATTTGACTCAACTCGGGGA
AACTTACCAGGTCCAGACATAATTAGGATTGACAGATTGATAGCTCTTTCTTGATTCTATGG
GTGGTGGTGCATGGCCGTTCTTAGTTGGTGGAGTGATTTGTCTGGTTAATTCCGTTAACGAAC
GAGACCTTAACCTGCTAAATAGTTACACATAATTCCGGTTATGTGGGCAACTTCTTA
>DQ500119.1 Pyrodinium bahamense var. compressum isolate G1
CAGGGCATCCATGTCTTGTAATTGGAATGAATGGAAATTAAACCTCTTTGTAAGTATCGATT
GGAGGGCAAGTCTGGTGCCAGCAGCCGCGGTAATTCCAGCTCCAATAGCGTATATTAAAGTT
GTTGCGGTTAAAAAGCTCGTAGTTGGATTTCTGCTAAGGGTGGCTGGTCCGCCCTCTGGGTG
AGTATCTGGCACAACCTGAGCATCTTTCTGGAGAACGTAACTGCACTTGACTGTGTGGTGCG
GTATCGAGAACTTTTACTTTGAGGAAATCAGAGTGTTTCAAGCAGGTGTTTGCCTTGAATAC
ATTAGCATGGAATAATAACATAGGACTTTGGTTCTATTTTGTTGGTTTCTAGAACGAAAATA
ATGATTAATGGGGATAGTTGGGGGCATTCGTATTTAATTGTCAGAGGTGAAATTCTTGGATT
TGTTGAAGACGGACTACTGCGAAAGCATTTGCCAAGGATGTTTTCATTGATCAAGAACGAAA
GTTAGGGGATCGAAGACGATCAGATACCGTCCTAGTCTTAACCATAAACCATGCCAACTAGA
GATTGGAGGCCGTTATTTGTACGATTCCTTCAGCACCTTATGAGAAATCGAAGTCTTTGGGTT
CCGGGGGGAGTATGGTCGCAAGGCTGAAACTTAAAGGAATTGACGGAAGGGCACCACCAGG
AGTGGAGCCTGCGGCTTAATTTGACTCAACACGGGGAAACTTACCAGGTCCAGACATAATTA
GGATTGACAGATTGATAGCTCTTTCTTGATTCTATGGGTGGTGGTGCATGGCCGTTCTTAGTT
GGTGGAGTGATTTGTCTGGTTAATTCCGTTAACGAACGAGACCTTAACCTGCTAAATAGTTA
CACATAATTCCGGTTATGTGGGCAACTTCTTA
>KX377183.1 Pyrodinium bahamense clone Pc-BRBD-1(1)-612
CAGGGCATCCATGTCTTGTAATTGGAATGAATGGAAATTAAACCTCTTTGTAAGTATCGATT
GGAGGGCAAGTCTGGTGCCAGCAGCCGCGGTAATTCCAGCTCCAATAGCGTATATTAAAGTT
GTTGCGGTTAAAAAGCTCGTAGTTGGATTTCTGCTAAGGGTGGCTGGTCCACCCTCTGGGTG
AGTATCTGGCACAACCTGAGCATCTTTCTGGAGAACGTAACTGCACTTGACTGTGTGGTGCG

GTATCGAGAACTTTTACTTTGAGGAAATCAGAGTGTTTCAAGCAGGTGTTTGCCTTGAATAC
ATTAGCATGGAATAATAACATAGGACTTTGGTTCTATTTTGTTGGTTTCTAGAACGAAAGTA
ATGATTAATAGGGATAGTTGGGGGCATTCGTATTTAATTGTCAGAGGTGAAATTCTTGGATT
TGTTGAAGACGGACTACTGCGAAAGCATTTGCCAAGGATGTTTTCATTGATCAAGAACGAAA
GTTAGGGGATCGAAGACGATCAGATACCGTCCTAGTCTTAACCATAAACCATGCCAACTAGA
GATTGGAGGCCGTTATTTGTACGATTCCTTCAGCACCTTATGAGAAATCGAAGTCTTTGGGTT
CCGGGGGGAGTATGGTCGCAAGGCTGAAACTTAAAGGAATTGACGGAAGGGCACCACCAGG
AGTGGAGCCTGCGGCTTAATTTGACTCAACACGGGGAAACTTACCAGGTCAAGACATAATTA
GGATTGACAGATTGATAGCTCTTTCTTGATTCTATGGGTGGTGGTGCATGGCCGTTCTTAGTT
GGTGGAGTGATTTGTCTGGTTAATTCCGTTAACGAACGAGACCTTAACCTGCTAAATAGTTA
CACATAATTCCGGTTATGCGGGCAACTTCTA
>KX377182.1 Pyrodinium bahamense clone Pc-BR-3(1)-808
CAGGGCATCCATGTCTTGTAATTGGAATGAATGGAAATTAAACCTCTTTGTAAGTATCGATT
GGAGGGCAAGTCTGGTGCCAGCAGCCGCGGTAATTCCAGCTCCAATAGCGTATATTAAAGTT
GTTGCGGTTAAAAAGCTCGTAGTTGGATTTCTGCTAAGGGTGGCTGGTCCGCCCTCTGGGTG
AGTATCTGGCACAACCTGAGCATCTTTCTGGAGAACGTAACTGCACTTGACTGTGTGGTGCG
GTATCGAGAACTTTTACTTTGAGGAAATCAGAGTGTTTCAAGCAGGTGTTTGCCTTGAATAC
ATTAGCATGGAATAATAACATAGGACTTTGGTTCTATTTTGTTGGTTTCTAGAACGAAAGTA
ATGATTAATAGGGATAGTTGGGGGCATTCGTATTTAATTGTCAGAGGTGAAATTCTTGGATT
TGTTGAAGACGGACTACTGCGAAAGCATTTGCCAAGGATGTTTTCATTGATCAAGAACGAAA
GTTAGGGGATCGAAGACGATCAGATACCGTCCTAGTCTTAACCATAAACCATGCCAACTAGA
GATTGGAGGCCGTTATTTGTACGATTCCTTCAGCACCTTATGAGAAATCGAAGTCTTTGGGTT
CCGGGGGGAGTATGGTCGCAAGGCTGAAACTTAAAGGAATTGACGGAAGGGCACCACCAGG
AGTGGAGCCTGCGGCTTAATTTGACTCAACACGGGGAAACTTACCAGGTCCAGACATAATTA
GGATTGACAGATTGATAGCTCTTTCTTGATTCTATGGGTGGTGGTGCATGGCCGTTCTTAGTT
GGTGGAGTGATTTGTCTGGTTAATTCCGTTAACGAACGAGACCTTAACCTGCTAAATAGTTA
CACATAACTCCGGTTATGTGGGCAACTTCTA
>KX377196.1 Pyrodinium bahamense clone Pc-MB-2-1112
CAGG_CATCCATGTCTTGTAATTGGAATGAATGGAAATTAAACCTCTTTGTAAGTATCGATTG
GAGGGCAAGTCTGGTGCCAGCAGCCGCGGTAATTCCAGCTCCAATAGCGTATATTAAAGTTG
TTGCGGTTAAAAAGCTCGTAGTTGGATTTCTGCTAAGGGTGGCTGGTCCGCCCTCTGGGTGA
GTATCTGGCACAACCTGAGCATCTTTCTGGAGAACGTAACTGCACTTGACTGTGTGGTGCGG
TATCGAGAACTTTTACTTTGAGGAAATCAGAGTGTTTCAAGCAGGTGTTTGCCTTGAATACA
TTAGCATGGAATAATAACATAGGACTTTGGTTCTATTTTGTTGGTTTCTAGAACGAAAGTAAT
GATTAATAGGGATAGTTGGGGGCATTCGTATTTAATTGTCAGAGGTGAAATTCTTGGATTTG
TTGAAGACGGACTACTGCGAAAGCATTTGCCAAGGATGTTTTCATTGATCAAGAACGAAAGT
TAGGGGATCGAAGACGATCAGATACCGTCCTAGTCTTAACCATAAACCATGCCAACTAGAG
ATTGGAGGCCGTTATTTGTACGATTCCTTCAGCACCTTATGAGAAATCGAAGTCTTTGGGTTC
CGGGGGGAGTATGGTCGCAAGGCTGAAACTTAAAGGAATTGACGGAAGGGCACCACCAGG
AGTGGAGCCTGCGGCTTAATTTGACTCAACACGGGGAAACTTACCAGGTCCAGACATAATTA
GGATTGACAGATTGATAGCTCTTTCTTGATTCTATGGGTGGTGGTGCATGGCCGTTCTTAGTT
GGTGGAGTGATTTGTCTGGTTAATTCCGTTAACGAACGAGACCTTAACCTGCTAAATAGTTA
CACATAATTCCGGTTATGTGGGCAACTTCTA
